# GLP-1 receptor agonists and cardiorenal outcomes in type 2 diabetes: an updated meta-analysis of eight CVOTs

**DOI:** 10.1186/s12933-021-01366-8

**Published:** 2021-09-15

**Authors:** Dario Giugliano, Lorenzo Scappaticcio, Miriam Longo, Paola Caruso, Maria Ida Maiorino, Giuseppe Bellastella, Antonio Ceriello, Paolo Chiodini, Katherine Esposito

**Affiliations:** 1 Division of Endocrinology and Metabolic Diseases, Department of Advanced Medical and Surgical Sciences, University of Campania “Luigi Vanvitelli”, Naples, Italy; 2 PHD Program of Translational Medicine, Department Advanced Medical and Surgical Sciences, University of Campania “Luigi Vanvitelli”, Naples, Italy; 3 Diabetes Unit, Department of Advanced Medical and Surgical Sciences, University of Campania “Luigi Vanvitelli”, Naples, Italy; 4 IRCCS MultiMedica, Milan, Italy; 5 Medical Statistics Unit, University of Campania “Luigi Vanvitelli”, Naples, Italy

**Keywords:** Cardiovascular outcome trials, Type 2 diabetes, GLP-1RA, Cardiorenal outcomes, Lixisenatide, Liraglutide, Semaglutide, Exenatide, Albiglutide, Dulaglutide, Oral semaglutide, Efpeglenatide

## Abstract

**Background:**

A meta-analysis is presented of cardiovascular outcome trials (CVOTs) comparing glucagon-like peptide-1 receptor agonists (GLP-1RA) versus placebo on cardiorenal outcomes in patients with type 2 diabetes mellitus (T2DM).

**Methods:**

We did an electronic search up to June 30, 2021, for eligible trials. We did a meta-analysis of available trial data using a random-effects model to calculate overall hazard ratios (HRs) and 95% CI (confidence intervals). We included data from 8 CVOTs and 60,080 patients (72.4% with established cardiovascular disease).

**Results:**

GLP-1RA reduced major cardiovascular events (MACE) by 14% (HR = 0.86, 95% CI 0.79–0.94, P = 0.006) with a non-significant heterogeneity between subgroups of patients with and without cardiovascular disease (P = 0.127). GLP-1RA also reduced the risk of cardiovascular death by 13% (P = 0.016), nonfatal stroke by 16% (P = 0.007), hospitalization for heart failure by 10% (P = 0.023), all-cause mortality by 12% (P = 0.012), and the broad composite kidney outcome by 17% (P = 0.012), which was driven by a reduction in macroalbuminuria only (HR = 0.74, 0.67–0.82, P < 0.001).

**Conclusions:**

GLP-1RA have moderate benefits on MACE, and also reduce hospitalization for heart failure and all-cause mortality; they also have robust benefits on reducing the incidence of macroalbuminuria.

## Introduction

A significant reduction in the incidence of major cardiovascular events (MACE) has been observed in patients with type 2 diabetes mellitus (T2DM) treated with glucagon-like peptide-1 receptor agonists (GLP-1RA), suggesting the possibility of cardioprotective actions for some molecules of the class [1, 2]. However, the amelioration of the cardiovascular outlook by GLP-1RA seems more prominent in patients with T2DM and pre-existing cardiovascular (CV) disease as compared with those with CV risk factors only [3–6]. In particular, the use of GLP-1RA, including lixisenatide, exenatide, liraglutide, semaglutide, albiglutide and dulaglutide, is associated with a significant 14% lower risk of MACE in patients with T2DM and history of CV disease, and with a nonsignificant 6% lower risk in those without history of CV disease [5]. However, the lack of significant statistical interaction between subgroups suggested caution in the net separation of MACE effect between patients with or without established CV disease. Accordingly, in patients with T2DM and established CV disease or multiple risk factors for CV disease, the American Diabetes Association (ADA) recommends a GLP-1RA with demonstrated cardiovascular benefit [liraglutide, albiglutide (removed from the market for business reasons), semaglutide, and dulaglutide] to reduce the risk of MACE [7]. In 2021, another cardiovascular outcome trial (CVOT) with the GLP-1RA efpeglenatide has been published, widening the range of the class of these drugs [8]. The aim of the present study was to examine the overall effect of GLP-1RA on cardiorenal efficacy to see whether these new findings could extend the generalizability of GLP-1RA trials. We synthesized data available from eight CVOTs in a meta-analysis to examine the efficacy of GLP-1RA on the main outcome MACE in patients with T2DM, with and without established CV disease, and to update their overall cardiorenal effects.

## Methods

### Search strategy and study selection

This systematic review was based on PRISMA (Preferred Reporting Items for Systematic Reviews and Meta-Analyses) guidelines [9]. The protocol has not been registered in any platform. We searched PubMed, EMBASE, the Cochrane Database of Systematic Reviews, and ClinicalTrials.gov. (http://www.clinicaltrials.gov) to identify all eligible trials with a primary outcome including cardiovascular mortality, non-fatal myocardial infarction, or non-fatal stroke (outcomes required by regulatory agencies for cardiovascular safety studies in diabetes), comparing the efficacy of GLP-1RA with that of placebo in adult patients with T2DM. The terms used for the research were ‘glucagon-like peptide-1 receptor agonists’, ‘GLP-1 agonist’, ‘lixisenatide’, ‘liraglutide’, ‘semaglutide’, ‘exenatide’, ‘albiglutide’, ‘dulaglutide’, ‘efpeglenatide’, ‘placebo’, ‘cardiovascular disease’, ‘cardiovascular risk factors’, and ‘randomized controlled trials’. The search was filtered to include only randomized controlled trials or meta-analyses of human data. Searches were done up until June 30, 2021. We excluded observational non-randomized studies, registries, ongoing trials without results, duplicate series, meta-analysis, abstracts, and oral communications. Data were extracted by D.G., L.S. and M.L., with conflicts over study inclusion resolved by consensus. We excluded trials if they were completed before the FDA guidance of 2008 [10], which was the starting point for all CVOTs examining the effect of GLP-1RA on MACE as primary endpoint. Inclusion criteria specified that cardiorenal outcomes of interest were included as part of the primary, secondary, exploratory or safety outcome.

### Data extraction and quality assessment

Results in trial reports (primary trial results and subsequent secondary publications), and their accompanying supplementary materials, were used as the primary source of information. The retrieved data included study characteristics, characteristics of patients, interventions, and outcome measures. The Cochrane Collaboration Risk-of-Bias tool was used for quality assessment of the RCTs [11], including sequence generation, allocation concealment, blinding, incomplete outcome data, and selective outcome reporting. Risk of bias was graded as unclear, high, or low.

### Statistical analysis

The primary efficacy outcome for this meta-analysis was the effect of GLP-1RA on the incidence of MACE. Moreover, the effect of GLP-1RA on MACE risk in patients with T2DM, with or without a history of CV disease at baseline, was a co-primary efficacy outcome. We did additional analyses for components of MACE (cardiovascular mortality, non-fatal myocardial infarction, non-fatal stroke), all-cause mortality, hospital admission for heart failure, the composite renal endpoint and incidence of new macroalbuminuria. HRs and 95% CI (confidence interval) for cardiorenal efficacy outcomes were synthesized. Heterogeneity between studies was evaluated by using the Cochran’s Q test, with P values of less than 0.10 representing significant heterogeneity. The proportion of variation in observed effects due to heterogeneity rather than sampling error was evaluated by using I^2^ index [12] and thresholds of I^2^ describing the degree of heterogeneity are 25% or lower (low), 26–50% (moderate), and greater than 50% (high). Pooled summary estimates were calculated according to random effects model using the empirical Bayes method that corresponds to Paule-Mandel method [13] with a Hartung-Knapp confidence interval adjustment [14], that was deemed necessary due to the small number of studies. Publication bias was not assessed as the number of trials was below ten. Data were analyzed using Stata, version 16.0 (Stata Corp., College Station, TX). All statistical tests were two-sided, and P values < 0.05 were regarded as significant.

## Results

### Search results and study characteristics

**Fig. 1:**
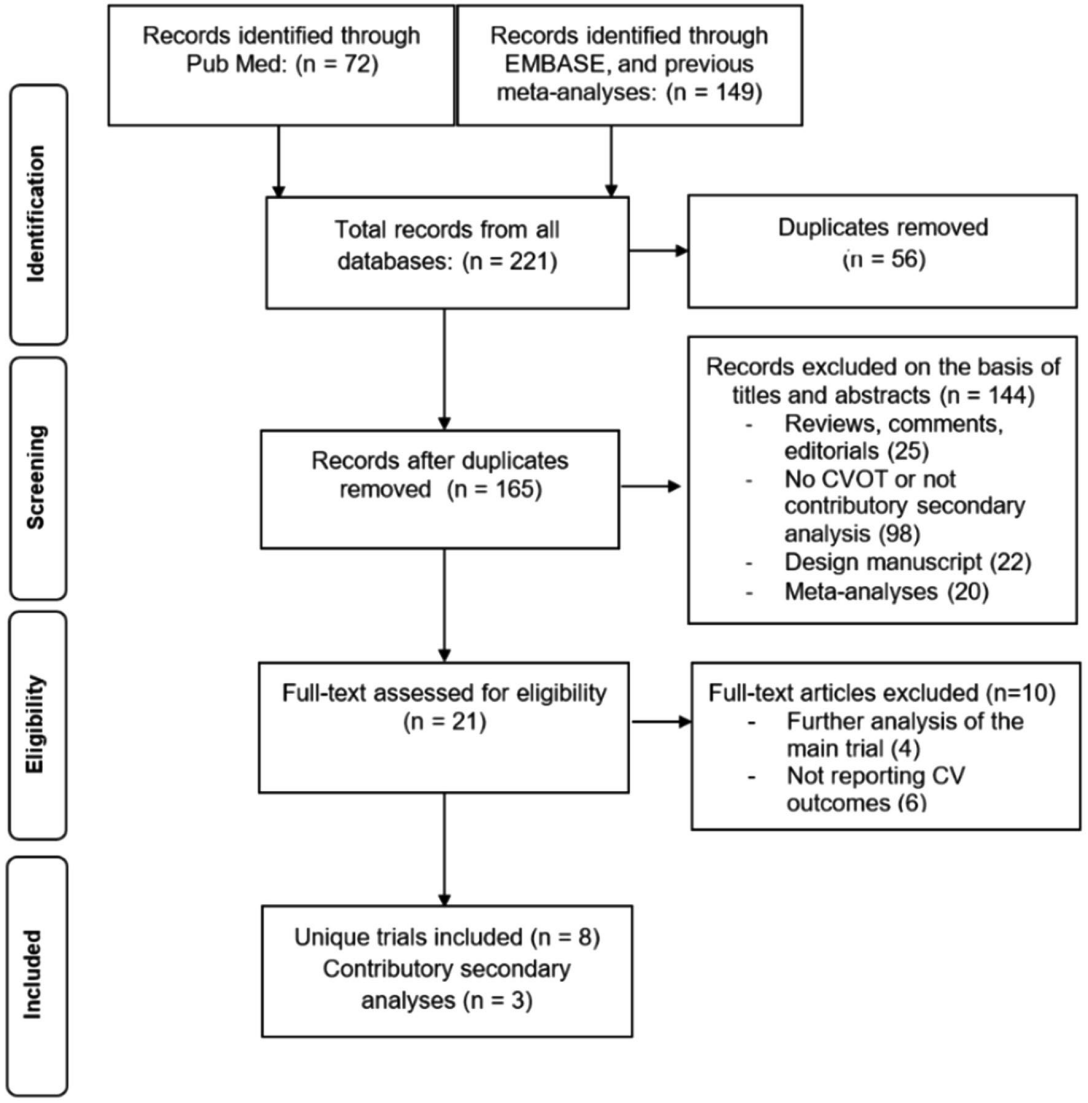
Process of studies’ selection

We identified a total of eight trials [8, 15–21] and three secondary analyses [22–24] from the same trials that were eligible for inclusion (Fig. 1). Their characteristics are summarized in Table 1. The participants were all adult (> 18 years old) patients with T2DM. All trials were multinational and sponsored by industry (two by Sanofi Aventis, three by Novo Nordisk, one by Eli Lilly, one by AstraZeneca, one by GlaxoSmithKline). The trials have been published between 2015 and 2021. All trials were of parallel-group, double-blind design, and their mean duration ranged from 1.3 to 5.4 years.

**Table 1:** Summary of CVOTs evaluating the cardioranal effects of GLP-1RAs in T2DM

Trial/year of Publication	Study drug/mean follow up (years)	Participants (n)	Age mean (years)	Male sex (n, %)	Participants with established CV disease (n, %)	History of heart failure (n, %)	eGFR < 60 ml/min per 1.73 m^2^ (n, %)
ELIXA	Lixisenatide	6068	60.3	3174 (69.3%)	6068 (100%)	1922 (20.3%)	1407 (23.2%)
2015	2.1 year						
LEADER	Liraglutide	9340	64.3	6003 (64.3%)	6764 (72.4%)	1667 (17.8%)	2158 (23.1%)
2016	3.8 year						
SUSTAIN-6	Semaglutide	3297	64.6	2002 (60.7%)	2735 (83%)	777 (23.6%)	939 (28.5%)
2016	3.1 year						
EXSCEL	Eenatide OW	14,752	62.0	9149 (62%)	10,792 (73.1%)	2389 (16.2%)	3191 (21.6%)
2017	3.2 year						
HARMONY	Albiglutide	9463	64.1	6569 (69.4%)	9463 (100%)	1922 (20.3%)	NR
2018	1.6 year						
REWIND	Dulaglutide	9901	66.2	5312 (53.7%)	3109 (31.4%)	853 (8.6%)	2199 (22.2%)
2019	5.4 year						
PIONEER 6	Semaglutide	3183	66.0	2176 (68.4%)	2695 (84.7%)	388 (12.2%)	856 (26.8%)
2019	1.3 year						
AMPLITUDE-O	Efpeglenatide	4076	64.5	2732 (67%)	3650 (89.6%)	737 (18.1%)	1287 (31.6%)
2021	1.8 year						

**Fig. 2:**
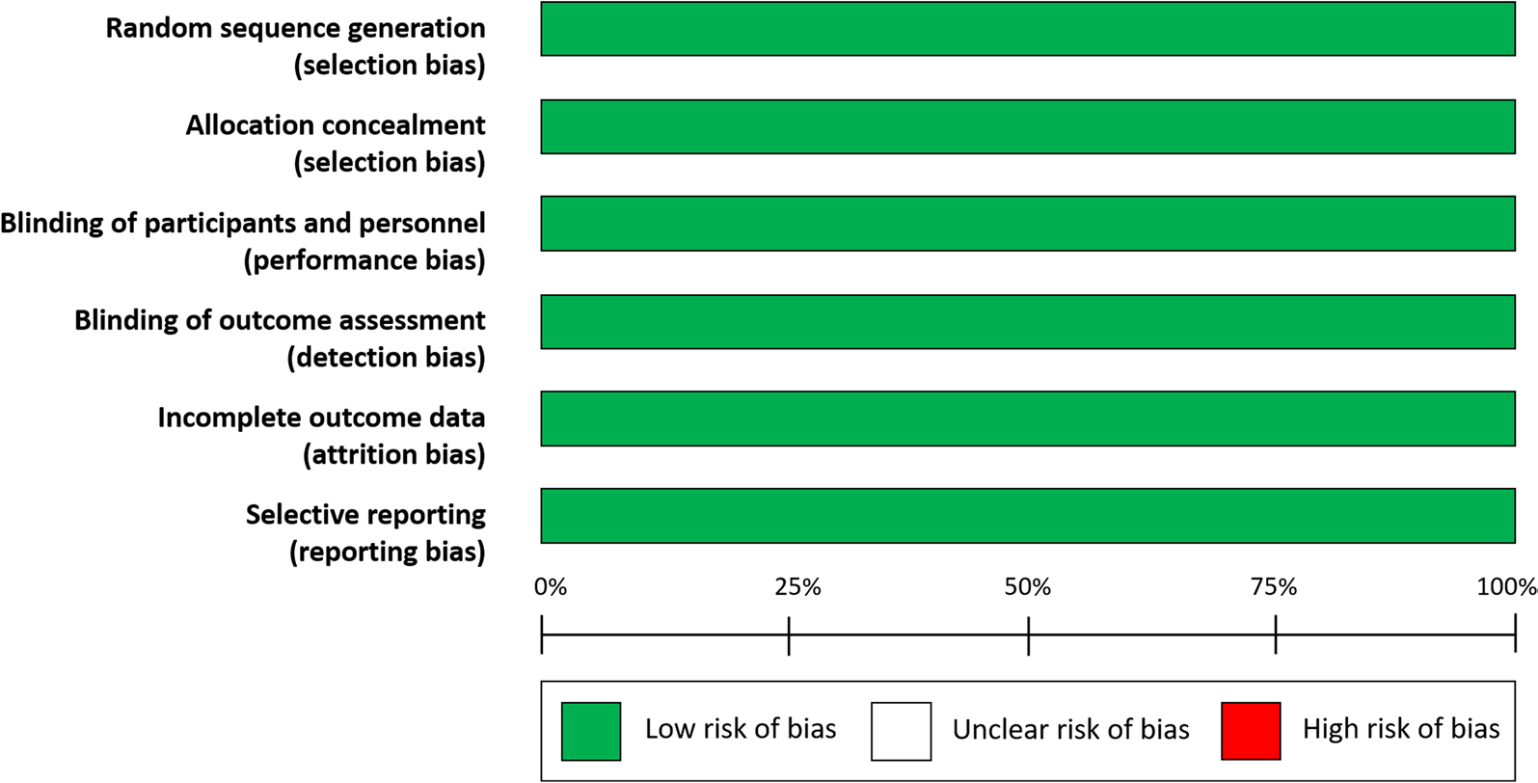
Cochrane risk of bias (graph) for the 8 trials

ELIXA compared lixisenatide to placebo in 6068 patients with T2DM who had suffered a recent acute coronary event [15]. LEADER compared liraglutide to placebo in 9340 patients with T2DM and known CV disease or CV risk factors only [16]. SUSTAIN-6 compared semaglutide to placebo in 3297 patients with T2DM and established CV disease or CV risk factors only [17]. EXSCEL compared extended release exenatide to placebo in 14,752 patients with T2DM and established CV disease or CV risk factors only [18]. HARMONY Outcomes compared albiglutide to placebo in 9463 patients with T2DM and known CV disease [19]. REWIND compared dulaglutide to placebo in 9901 patients with T2DM and previous CV events or CV risk factors only [20]. PIONEER 6 compared oral semaglutide to placebo in 3183 patients with T2DM and established CV disease or CV risk factors only [21]. AMPLITUDE-O compared efpeglenatide to placebo in 4076 adults with T2DM and previous CV events or CV risk factors only [8]. The primary outcome for LEADER, SUSTAIN-6, EXSCEL, HARMONY Outcomes, REWIND, PIONEER 6 and AMPLITUDE-O was a three-point MACE, whereas ELIXA used a four-point MACE, including also hospital admission for unstable angina. Characteristics of trials and patients are reported, respectively, in Table 1. The populations studied ranged in size from 3297 (SUSTAIN-6) to 14,752 (EXSCEL), were of similar age (mean age was 64.0 ± 1.97 years), 37,117 were male (62.8%), and the median duration of follow-up ranged from 1.3 to 5.4 years. According to the Cochrane Collaboration’s tool for assessing risk of bias, there was no major risk of bias in any study (Fig. 2).

### Outcomes

**Fig. 3:**
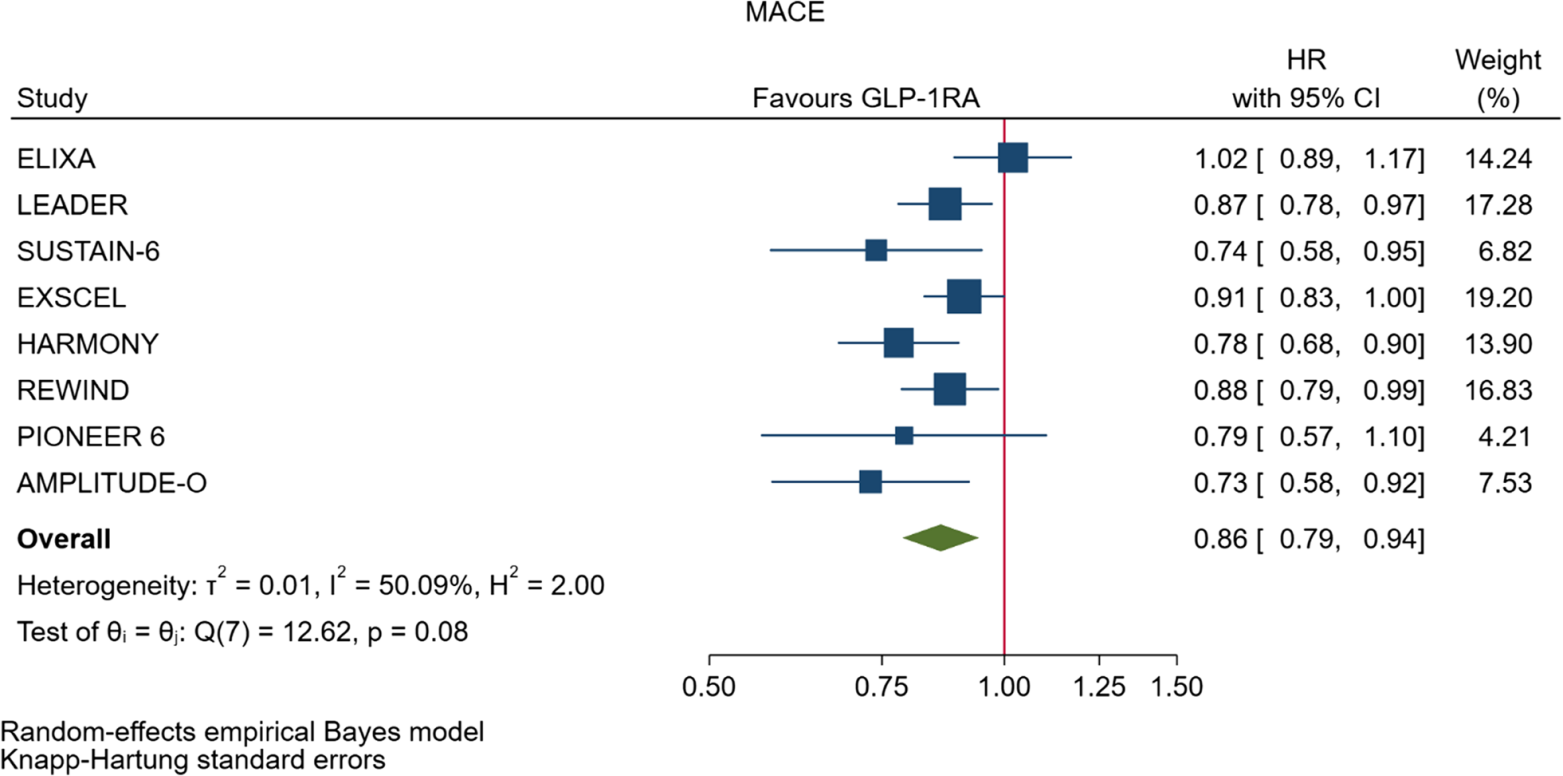
Forest plots of meta-analysis of the eight CVOTs with GLP-1RA on MACE. The results are expressed as HR (hazard ratio)

**Fig. 4:**
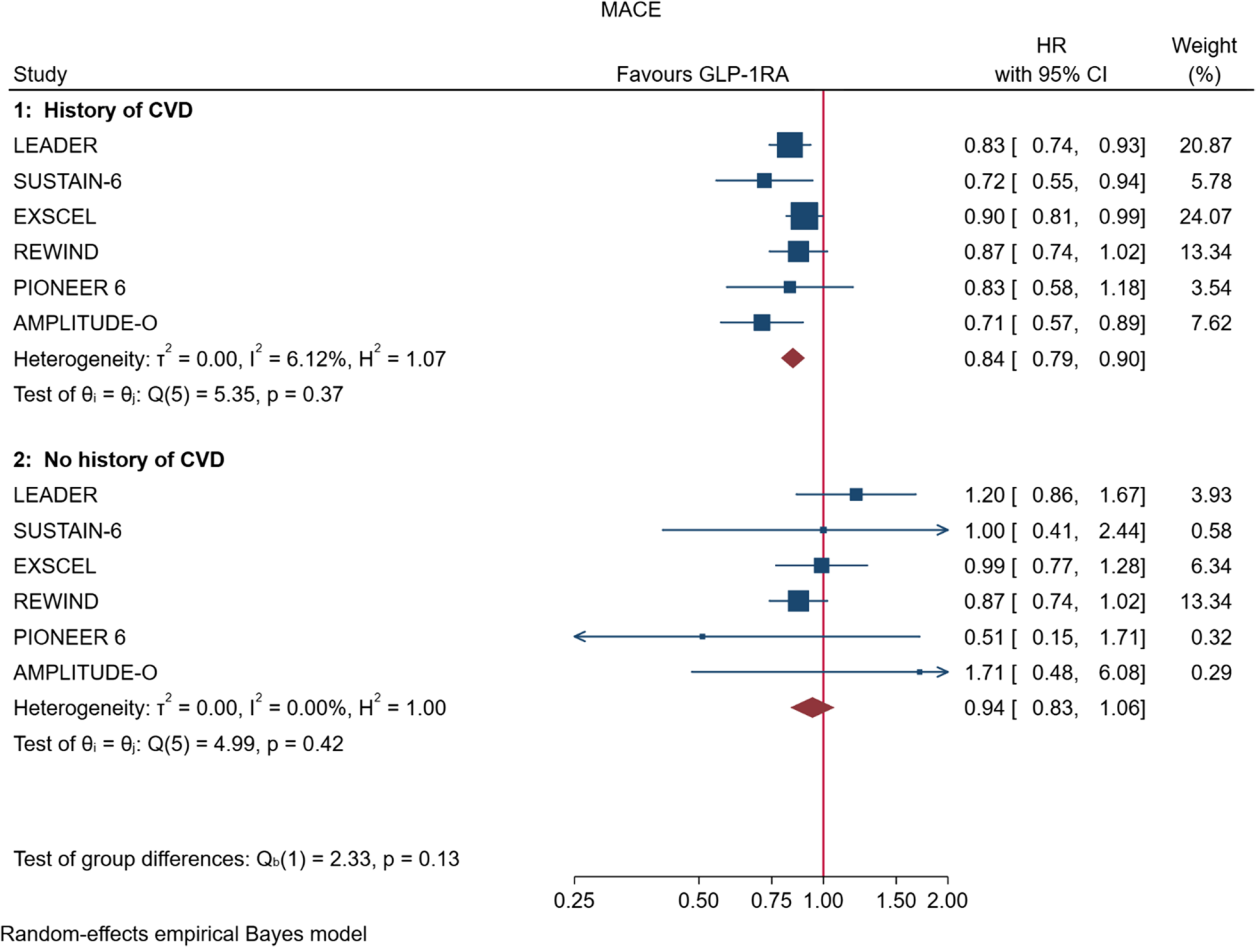
Forest plots of meta-analysis of the six CVOTs with GLP-1RA on MACE, in patients with history of CV disease (top) or in patients without such a history (bottom)

In the overall analysis, the risk of MACE was reduced by 14% (HR = 0.86, 95% CI, 0.79–0.94, P = 0.006) compared with placebo with no significant heterogeneity between trials (I^2^ = 50.0%, P = 0.08) (Fig. 3, Table 2). Figure 4 shows the forest plots of the six CVOTs that reported the evaluation of MACE risk as a sub-analysis of patients with T2DM divided according to the presence or absence of CV disease at baseline, respectively. In these CVOTs (LEADER, SUSTAIN-6, EXSCEL, REWIND, PIONEER 6, AMPLITUDE-O), the percentage of patients with CVD at baseline was 72.4%; compared with placebo, treatment with GLP-1RA was associated with a 16% and 6% lower risk of MACE in patients with or without preexisting CV disease, respectively (Table 2). The heterogeneity between subgroups was not significant (P = 0.127) suggesting that patients with or without a history of CVD behave as a whole population.

**Table 2:** Results of planned meta-analyses with random effects

Outcome	Trials (n)	Estimate (HR)	95% CI	P value of HR	I^2^ (%)	P value of I^2^
MACE						
All	8	0.86	0.79–0.94	0.006	50.0	0.080
Prior CVD	6	0.84	0.79–0.90	< 0.001	6.1	0.370
No prior CVD	6	0.94	0.83–1.06	0.330	0.0	0.420
CV mortality	8	0.87	0.78–0.96	0.016	18.7	0.330
Non-fatal MI	8	0.91	0.81–1.01	0.078	34.6	0.170
Non-fatal stroke	8	0.84	0.76–0.94	0.007	0.0	0.580
Heart failure	8	0.90	0.83–0.98	0.023	0.0	0.670
All-cause mortality	8	0.88	0.80–0.96	0.012	26.3	0.350
Renal endpoints	6	0.83	0.73–0.94	< 0.012	36.5	0.280
New macro	6	0.74	0.67–0.82	< 0.001	11.0	0.370

*Macro* macroalbuminuria

**Fig. 5:**
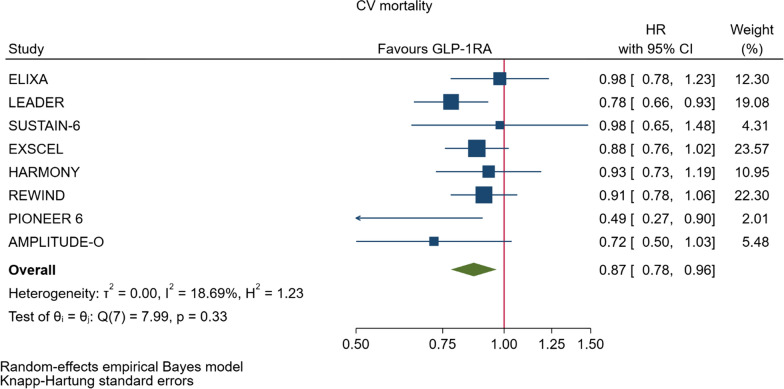
Forest plots of meta-analysis of the eight CVOTs with GLP-1RA on CV mortality

**Fig. 6:**
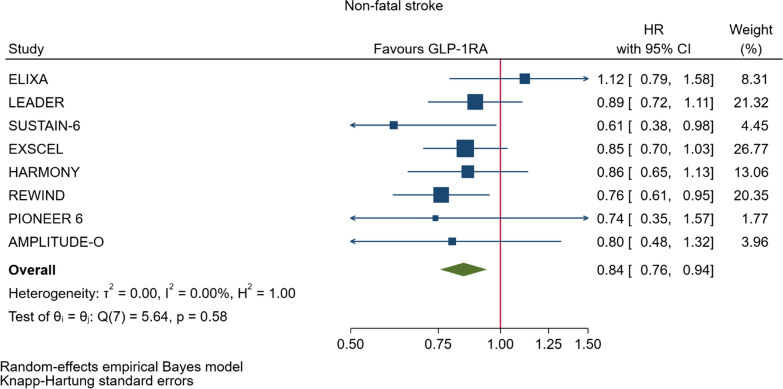
Forest plots of meta-analysis of the eight CVOTs with GLP-1RA on nonfatal stroke

**Fig. 7:**
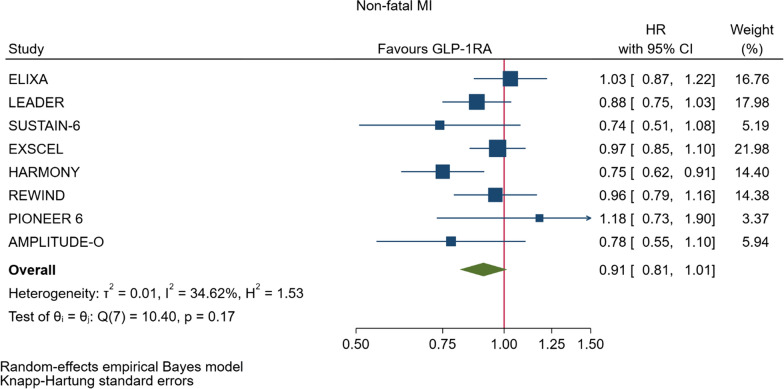
Forest plots of meta-analysis of the eight CVOTs with GLP-1RA on nonfatal myocardial infarction

**Fig. 8:**
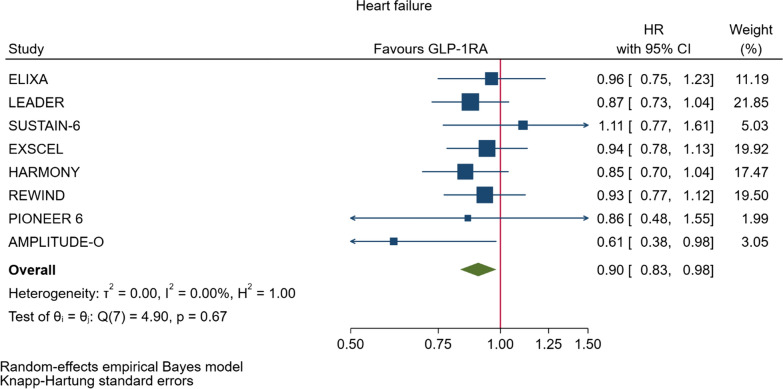
Forest plots of meta-analysis of the eight CVOTs with GLP-1RA on hospitalization for heart failure

**Fig. 9:**
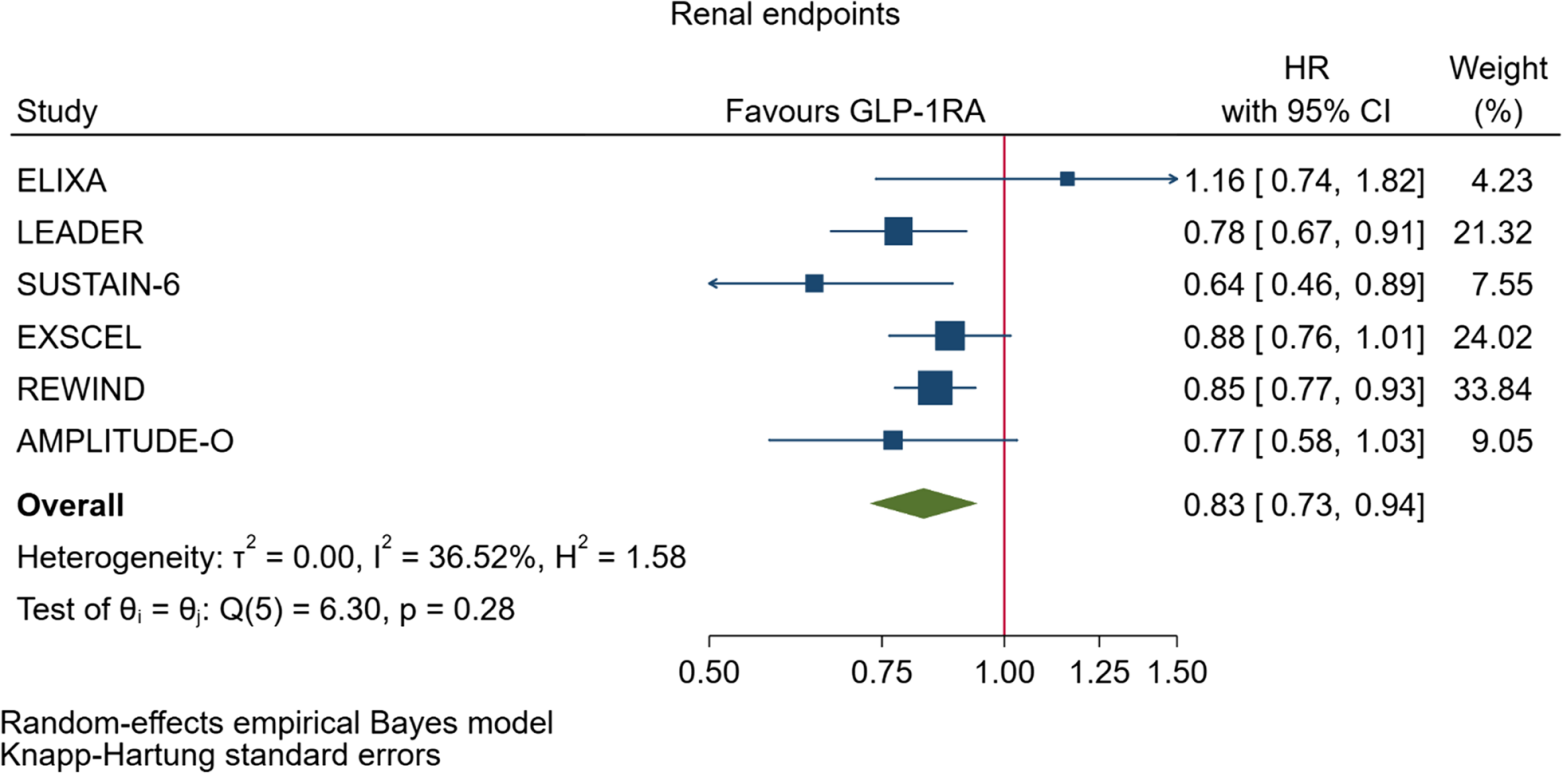
Forest plots of meta-analysis of the eight CVOTs with GLP-1RA on composite renal endpoint

**Fig. 10:**
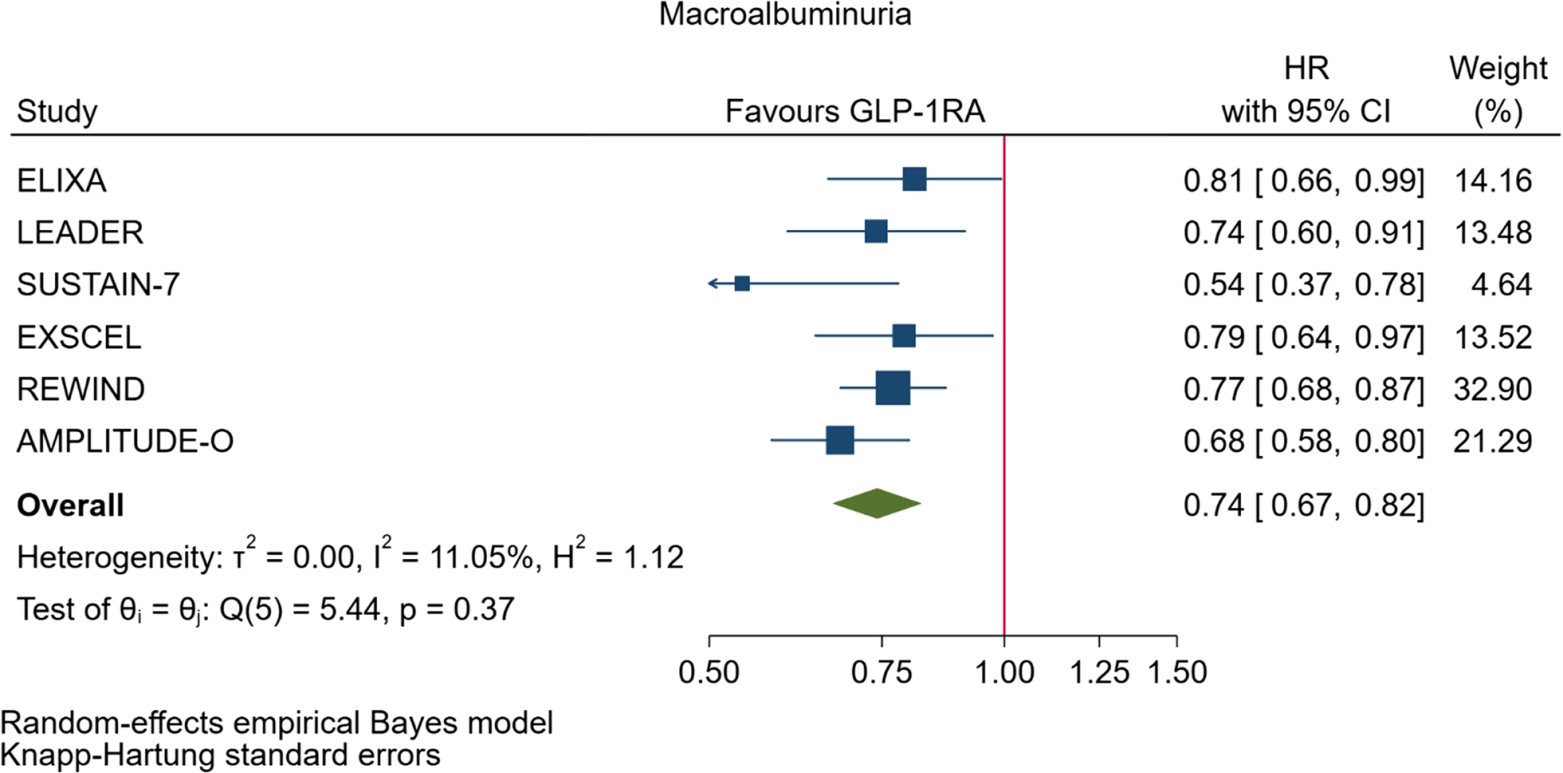
Forest plots of meta-analysis of the eight CVOTs with GLP-1RA on incidence of new macroalbuminuria

As far as the three MACE components are concerned, the use of GLP-1RA was associated with 13% reduced risk of cardiovascular mortality (HR = 0.87, Fig. 5, Table 2), and 16% reduced risk of non-fatal stroke (HR = 0.84, Fig. 6, Table 2). However, non-fatal myocardial infarction showed a nonsignificant 9% decrease (HR = 0.91, Fig. 7, Table 2). The risks of hospitalization for heart failure (HR = 0.90, Fig. 8, Table 2) and of all-cause mortality (HR = 0.88, Table 2) were also significantly reduced. In six CVOTs, GLP1-RA reduced the risk of the broad composite kidney outcome by 17% (HR = 0.83, Fig. 9, Table 2), which appeared to be driven by a reduction in macroalbuminuria only (HR = 0.74, Fig. 10, Table 2). The estimates of both renal endpoints were associated with no significant heterogeneity (Table 2).

## Discussion

The results of the current meta-analysis, that included 60,080 patients with T2DM, 14,804 of whom without established CV disease, demonstrate that GLP-1RA reduce the risk of MACE by 14% in the overall diabetic population, with an apparently greater effect in patients with known CV disease as compared with those without established CV disease (16% vs 6% reduction, respectively). However, the lack of significant statistical interaction between subgroups (P = 0.127) suggests that addition of the AMPLITUDE-O trial has not changed the previous scenario [5], stressing that the net separation on MACE effect between patients with or without established CV disease may be more nuanced than suggested by previous meta-analyses [3]. This interpretation is further supported by the finding that heterogeneity was not significant in the primary analysis (MACE) and practically null in the secondary analysis (MACE subgroups). GLP-1 receptor agonists also reduce the three components of MACE, including CV mortality (reduced by 13%), nonfatal stroke (reduced by 16%) and nonfatal myocardial infarction (reduced by 9%, although the level of significance was not reached).

Compared with other CVOTs involving GLP-1RA, participants in the AMPLITUDE-O trial had the longest duration of diabetes (15 years), lowest mean eGFR (72 ml/min/1.73 m^2^) with a higher percentage (32%) of people with renal disease (eGFR 25–60 mL/min), highest glycosilated hemoglobin (8.9%), and highest percentage of insulin use (62%) or sodium–glucose transporter-2 inhibitors (SGLT-2i) (15%) at baseline, thus identifying a more fragile population. The addition of data from AMPLITUDE-O trial to the seven-trial meta-analysis [5] showed that patients with type 2 diabetes who received GLP-1 agonists had a decreased risk of MACE and decreased individual components (stroke more than myocardial infarction), independently of the structure of these drugs (exendine-4 based or human analogs).

GLP-1RA significantly reduced the risk of heart failure by 10% and all-cause mortality by 12%. The reduction of risk for hospitalization for heart failure was quite like that recorded in previous meta-analysis [1, 5], with the only exception that AMPLITUDE-O is the only trial associated with a significant reduction of HF risk (39% reduction). Although a modest benefit of GLP-1RA in preventing HF hospitalizations has emerged, this finding deserves to be confirmed in mechanistic studies. The atherosclerotic benefits provided by GLP-1RA, which reflect the improvement of glycated hemoglobin level, blood pressure, albumin-to-creatinine ratio and other favorable hemodynamic effects, may be responsible, at least in part, for this finding. However, across CVOTs with GLP-1RA, details about heart failure were often incomplete and not standardized. Finally, none of the trials included HF in the primary composite outcome, although all trials prospectively adjudicated HF events as secondary outcomes. By contrast, the reduction of all-cause mortality by GLP-1RA was significant in three trials (LEADER with liraglutide, EXSCEL with exenatide, and PIONEER 6 with semaglutide).

GLP1-RA reduced the risk of the broad composite kidney outcome significantly by 17% which was only driven by a reduction in macroalbuminuria (26% reduction). In absolute terms, reduction of new-onset macroalbuminuria was the greatest efficacy outcome observed with the use of GLP-1RA so far investigated.

Our results are in line with a recent network meta-analysis including 764 trials and 421,346 patients [25] which demonstrated that use of GLP-1RA reduces all cause and cardiovascular mortality, non-fatal myocardial infarction, non-fatal stroke, and kidney failure, with a substantial benefit on non-fatal stroke over SGLT-2 inhibitors and notable differences in the tolerability profile. GLP-1RA may thus confer benefits when used in individuals with established cardiovascular disease (mainly on atherosclerotic basis) or chronic kidney disease who accept injections.

The cardiorenal benefits by GLP-1RA may be partially mediated by their effects on glycated hemoglobin level, blood pressure, and other conventional CV risk factors [26, 27]; a meta-regression analysis suggested a linear relation between the degree of lowering of glycated hemoglobin level and the hazard of MACE or stroke with GLP-1RA [28]. The observed reduction of risk of a composite renal outcome event also suggests that GLP-1RA may also have salutary endothelial and microvascular effects [29]. Other possible mechanisms include antiinflammatory, antifibrotic, antiatherosclerotic, vasodilatory, and other hemodynamic effects [30].

The strengths of this meta-analysis are the inclusion of all GLP-1RA CVOTs published until June 2021, the very large number of participants, the use of MACE as the main endpoint, the high quality of all trials which minimizes the risk of bias, and the absence of heterogeneity for all the endpoints considered. Several limitations should also be noted. The current is a meta-analysis of trial data, and we acknowledge the superiority of patient-level meta-analysis. Use of aggregate data limits the possibility to investigate subgroups of patients and suggest caution about the observed differences in treatment effects between subgroups (with or without pre-existing CV disease). We did not explore the effects of GLP-1RAs on the examined endpoint according to gender. There is evidence from a previous meta-analysis that GLP-1RA confer a similar reduction in MACE in both sexes, whereas SGLT-2 inhibitors reduce the risk of MACE more in men than in women [31]. Women are generally underrepresented in CVOTs, leading to a potential inadequate statistical power. In addition, the exact inclusion/exclusion criteria and definitions of endpoints differed slightly among the included trials: this is particularly evident for the renal endpoints. Trials with head-to-head comparison would be necessary to demonstrate possible superiority of a drug within the GLP-1RA class. On the other hand, a recent network meta-analysis [32] indirectly compared CV effects among different GLP-1RA in patients with T2DM and did not find any significant difference between GLP1RA in reducing death from any cause, MI and stroke events. However, the ranking results showed that oral semaglutide had the highest probability to be ranked first (> 90%) in reducing CV death and death from any cause, while once weekly semaglutide had the highest probability to be ranked first in reducing MI and stroke events.

## Conclusions

GLP1-RA reduce the risk of MACE by 14% in eight CVOTs with 60,080 patients with T2DM over a period ranging from 1.3 to 5.4 years. Although these data show a favorable risk–benefit profile for GLP-1RA, there are differences between individual drugs with respect to their effect on cardiorenal outcomes in the separate trials. Because the prevalence of CVD in the population with T2DM is around 32% [33], at least one fourth of the average patient with T2DM is possible a candidate for the use of the GLP-1RA, or sodium-glucose transporter-2 inhibitors [34], to improve the MACE outcome.

## Data Availability

All data generated or analyzed during this study are included in this published article.

## Abbreviations

ADA: American Diabetes Association
CV: Cardiovascular
CVOTs: Cardiovascular outcome trials
GLP-1RA: Glucagon-like peptide-1 receptor agonists
HR: Hazard ratios
MACE: Major cardiovascular events
SGLT-2i: Sodium–glucose transporter-2 inhibitors
T2DM: Type 2 diabetes mellitus
